# An Optimization-Based Locomotion Controller for Quadruped Robots Leveraging Cartesian Impedance Control

**DOI:** 10.3389/frobt.2020.00048

**Published:** 2020-04-24

**Authors:** Guiyang Xin, Wouter Wolfslag, Hsiu-Chin Lin, Carlo Tiseo, Michael Mistry

**Affiliations:** ^1^School of Informatics, Institute of Perception, Action and Behaviour, The University of Edinburgh, Edinburgh, United Kingdom; ^2^Edinburgh Centre for Robotics, The University of Edinburgh, Edinburgh, United Kingdom; ^3^School of Computer Science, Department of Electrical and Computer Engineering, McGill University, Montreal, QC, Canada

**Keywords:** locomotion controller, projected inverse-dynamics, quadratic programming, impedance control, quadruped robots

## Abstract

Quadruped robots require compliance to handle unexpected external forces, such as impulsive contact forces from rough terrain, or from physical human-robot interaction. This paper presents a locomotion controller using Cartesian impedance control to coordinate tracking performance and desired compliance, along with Quadratic Programming (QP) to satisfy friction cone constraints, unilateral constraints, and torque limits. First, we resort to projected inverse-dynamics to derive an analytical control law of Cartesian impedance control for constrained and underactuated systems (typically a quadruped robot). Second, we formulate a QP to compute the optimal torques that are as close as possible to the desired values resulting from Cartesian impedance control while satisfying all of the physical constraints. When the desired motion torques lead to violation of physical constraints, the QP will result in a trade-off solution that sacrifices motion performance to ensure physical constraints. The proposed algorithm gives us more insight into the system that benefits from an analytical derivation and more efficient computation compared to hierarchical QP (HQP) controllers that typically require a solution of three QPs or more. Experiments applied on the ANYmal robot with various challenging terrains show the efficiency and performance of our controller.

## 1. Introduction

Recent improvements in mobile robotics indicate that it is possible to achieve autonomous inspection and maintenance of critical industrial infrastructure in extreme environments (e.g., off-shore plants and nuclear sites) within the next decade. The automation of such processes will reduce their economic cost and increase the life quality of the operators that can be moved away from operational hazards. Legged robots are the most promising among ground robots for locomotion flexibility, being able to surpass a varied set of obstacles. However, this comes at the expense of reduced stability compared to wheeled robots. Quadruped robots offer a balance between efficiency and stability. Algorithms that can generate precise but compliant motions while satisfying physical feasibility are required to control quadruped robots with a large number of degrees of freedom. On the other hand, a trade-off strategy is also required to coordinate multiple tasks and constraints.

A good example for legged locomotion is the management of the contact forces that need to provide sufficient support to the body while not violating the non-slipping conditions determined by the friction cones. An intuitive control technique that can deal with these problems is the Virtual Model Controller (VMC) (Pratt et al., 2001), which has been applied to state of the art legged prototypes (Boaventura et al., 2012; Gehring et al., 2013). The VMC determines the contact forces based on the centroidal momentum model according to desired motions, and then maps these forces into joint torques. Meanwhile, the swing legs are controlled using joint PD controllers tracking a desired reference trajectory. Therefore, VMC controls swing legs and supporting legs separately and is not a whole-body controller.

Another widely used control framework is the fully optimization-based control (De Lasa et al., 2010; Saab et al., 2013). This framework relies on solving an optimization problem (usually quadratic) to minimize a cost function. The dynamic model and desired end-effector motions are introduced as equality constraints, while friction cones, torques, and joint limitations are inequality constraints. The main benefit of the optimization-based control is the integration of the operational space tasks and the joint space dynamics in a single problem. Furthermore, its formulation can be extended to solve multiple tasks, which can be implemented using either weighted optimization or strictly hierarchical optimization (Hutter et al., 2014). Two popular implementations are the Hierarchical Quadratic Programming (HQP) and the Weighted Quadratic Programming (WQP). HQP uses a strictly prioritized control framework based on null-space projectors; meanwhile, WQP relies on tunable weight matrices. Despite the fact that HQP solves two or more QPs, it is normally preferred to WQP because it does not require tuning.

The QP-schemes outlined above optimize for joint accelerations, contact forces, and joint torques, but only joint torques are eventually used for control. Since only torques are needed to control the robot, the original optimization formulation can be simplified by reducing optimization variables by considering reducing computation complexity as well. In Bellicoso et al. (2016), the QR decomposition proposed in Mistry et al. (2010) is employed to eliminate contact forces from dynamic equations so that optimization variables are reduced to two vectors. In Mansard (2012), a projector considering the consistency of the inertia matrix is proposed to separate the dynamic equations, and the optimization variables are finally reduced to two vectors. A straightforward decomposition is presented in Herzog et al. (2016) that directly splits six rows corresponding to six dimensional floating bases from the whole dynamic equation, thus removing actuation torques from optimization variables. By contrast, joint torques are the decision variables for the proposed control technique in this paper, and only one QP needs to be solved.

For optimization-based approaches, operational tasks are bounded to joint space dynamics by optimization constraints. Consequently, tuning parameters, such as impedance gains require several trials. A seminal control technique called operational space control (OSC), presented first in Khatib (1987), provides us with the analytical solution to derive joint torques from desired operational tasks. OSC has been extended to hierarchical OSC by iterative null-space projection for legged robots (Sentis and Khatib, 2006; Sentis et al., 2010; Lee et al., 2016). In Mistry and Righetti (2012), the orthogonal projector proposed by Aghili (2005) is applied to OSC with the consideration of reducing complexity. Subsequently, in Lin et al. (2018) and Xin et al. (2018), we extend that approach to involve the analytical Cartesian impedance controller proposed in Albu-Schaffer et al. (2003) and QP optimization. This paper is a further extension of our previous works under the framework of projected inverse dynamics.

The formulation proposed in this manuscript is a synergistic integration between the analytical Cartesian impedance controller and the QP. The method enables compensation of model errors while operating a trade-off strategy between multiple constraints, thus improving the locomotion capability traversing steep and slippery terrains. Specifically, the contributions of this paper come from combining the following components in a single control pipeline:

- Analytical Cartesian impedance control, based on the orthogonal projected inverse-dynamics, which allows us to analyze a legged robot using a mass-spring-damper model against disturbances.
- Perturbation estimation using the impedance behavior to evaluate disturbances including external forces and model errors.
- QP optimization that performs a trade-off strategy to relax trajectory tracking when feasibility constraints are close to violation, which is critical when walking in extreme environments.

**Figure 1:**
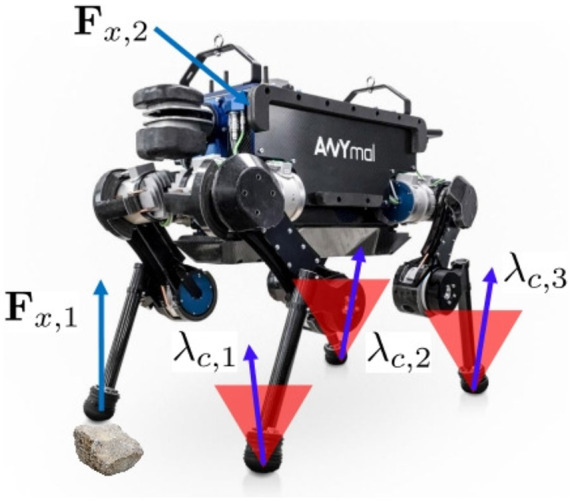
A quadruped robot, ANYmal, with potential external disturbances ***F***_*x*_ and contact forces **λ**_*c*_ within friction cones during static walking.

Finally, the proposed pipeline and controller are extensively validated on the torque controllable quadruped robot ANYmal (see Figure 1) in various scenarios.

## 2. Problem Formulation

In this section we consider the general problem of constrained inverse-dynamics for quadruped robots, including a discussion of the controllability of quadruped robots. Then we present the projected inverse-dynamics to deal with constrained dynamics, which we will use to derive our locomotion controller for quadruped robots.

### 2.1. Dynamic Model of Quadruped Robots

The rigid-body dynamics equation of a floating base system under contact constraints is

(1) $$\begin{array}{r} \text{M}\ddot{\text{q}}+\text{h}=\text{B}\tau +{\text{J}}_{c}^{⊤}\lambda \end{array}$$

where $\text{q}={\begin{array}{cc} {}[{\text{x}}_{b}^{⊤} & \theta^{⊤}] \end{array}}^{⊤}$ denotes the vector of actuated joint positions (***θ*** ∈ ℝ^*n*^) and floating base position and orientation (**x**_*b*_ ∈ *SE*(3)), **M** ∈ ℝ^(*n*+6) × (*n*+6)^ is the inertia matrix, **h** ∈ ℝ^*n*+6^ is the generalized vector containing Coriolis, centrifugal and gravitational effects, ***τ*** ∈ ℝ^*n*+6^ is the vector of torques, ${\text{J}}_{c}\in {\mathbb{R}}^{k\times \left(n+6\right)}$ is the constraint Jacobian that describes *k* linearly independent constraints, **λ** ∈ ℝ^*k*^ is the generalized constraint force vector that enforces the condition of ${\text{J}}_{c}\ddot{\text{q}}+{\overset{∙}{\text{J}}}_{c}\overset{∙}{\text{q}}=\text{0}$ (or ${\text{J}}_{c}\overset{∙}{\text{q}}=\text{0}$), and

(2) $$\begin{array}{r} \text{B}=\left[\begin{array}{cc} 0_{6} & 0\\ 0 & {\text{I}}_{n} \end{array}\right] \end{array}$$

is the projector into actuated joint space with *n* dimensional identity matrix **I**_*n*_.

It should be noted that **J**_*c*_ = [**J**_*c*_*b*__ **J**_*c*_*s*__], whereby ${\text{J}}_{c_{b}}\in {\mathbb{R}}^{k\times 6}$ and ${\text{J}}_{c_{s}}\in {\mathbb{R}}^{k\times n}$. **J**_*c*_*b*__ encodes the effect of contact constraints on the floating base, with its rank providing direct insights into the controllability properties of the robots (Mistry et al., 2008). For quadruped robots, with the assumption of point contacts, there is *k* = 3 × *n*_*c*_ where *n*_*c*_ denotes the number of legs in stance. The case of *rank*(**J**_*c*_*b*__) < 6 (such as during a trotting gait with only two stance legs, and static walking with three stance feet in a line) is called truly underactuated (Hutter et al., 2014) which means there are not enough contact constraints to independently control the six unactuated base coordinates **x**_*b*_. The case of *rank*(**J**_*c*_) − *rank*(**J**_*c*_*b*__) > 0 is called overconstrained which means we can use redundancy to control the constraint/internal forces. Static walking (except with co-linear feet) is a typical overconstrained case, as *rank*(**J**_*c*_) − *rank*(**J**_*c*_*b*__) = 9 − 6 = 3 with one swing leg. The proposed controller incorporating QP aims to control the internal forces to satisfy contact constraints. Moreover, one should note that we cannot independently generate the contact forces for both static walking and trotting gait because of *rank*(**J**_*c*_) − *rank*(**J**_*c*_*b*__) < *rank*(**J**_*c*_).

### 2.2. Projected Inverse Dynamics

The dynamics Equation (1) can be divided into two subspaces: the motion space where the acceleration can be chosen freely, and the constraint space where the acceleration is fixed and constraint forces can be chosen. Working with the two subspaces allows us to satisfy contact constraints independently of joint motion. Paper (Aghili, 2005) proposes an orthogonal projector derived only from kinematic parameters without consideration of constraint dynamic consistency, leading to a much simplified solution compared to Sentis et al. (2010). The employment of this orthogonal projector is the key for analytical Cartesian impedance control and optimization variable reduction.

The constraint equation ${\text{J}}_{c}\overset{∙}{\text{q}}=\text{0}$ implies that any admissible generalized velocity must lie in the null space of matrix **J**_*c*_, i.e., $\overset{∙}{\text{q}}\in N\left({\text{J}}_{c}\right)$. We can use the pseudoinverse of **J**_*c*_ to compute the orthogonal null space projector **P** as $\text{P}=\text{I}-{\text{J}}_{c}^{+}{\text{J}}_{c}$, such that $R\left(\text{P}\right)=N\left({\text{J}}_{c}\right)$, $\text{P}\overset{∙}{\text{q}}=\overset{∙}{\text{q}}$, $\text{P}{\text{J}}_{c}^{⊤}=\text{0}$, and **P**^2^ = **P**^⊤^ = **P**. Since the vectors lying in $N\left({\text{J}}_{c}\right)$ denote the motions of torso and any end-effector in the air, we call $N\left({\text{J}}_{c}\right)$ the motion space. It should noted that any vector could be projected into motion space by pre-multiplying with the projection matrix **P**. Then the complimentary projection **I** − **P** projects vectors into the constraint space $N{\left({\text{J}}_{c}\right)}^{⊥}=R\left({\text{J}}_{c}^{⊤}\right)$. Subsequently, we can split Equation (1) into two equations of two orthogonal spaces (motion space and constraint space) using the above two projectors:

(3) $$\begin{array}{r} \text{P}\text{M}\ddot{\text{q}}+\text{P}\text{h}=\text{P}\text{B}\tau =\tau_{m} \end{array}$$

(4) $$\begin{array}{r} \left(\text{I}-\text{P}\right)\left(\text{M}\ddot{\text{q}}+\text{h}\right)=\left(\text{I}-\text{P}\right)\text{B}\tau +{\text{J}}_{c}^{⊤}\lambda =\tau_{c}+{\text{J}}_{c}^{⊤}\lambda \end{array}$$

and then torque command ***τ*** = ***τ***_*m*_ + ***τ***_*c*_. Constraint torques ***τ***_*c*_ in Equation (4) do not contribute to the motion of the system, but system motion will affect contact forces due to $\ddot{\text{q}}$ in Equation (4). We wish to invert **PM** in Equation (3) to solve $\ddot{\text{q}}$, and then substitute that result into (4) to determine contact forces. However, **PM** is not invertible due to the rank deficiency of **P**. The contact constraint dictates that $\left(\text{I}-\text{P}\right)\overset{∙}{\text{q}}=0$, and its derivative

(5) $$\begin{array}{r} \left(\text{I}-\text{P}\right)\ddot{\text{q}}=\overset{∙}{\text{P}}\overset{∙}{\text{q}} \end{array}$$

Substituting Equation (5) into Equation (3) yields

(6) $$\begin{array}{r} {\text{M}}_{c}\ddot{\text{q}}+\text{Ph}-\overset{∙}{\text{P}}\overset{∙}{\text{q}}=\tau_{m} \end{array}$$

where **M**_*c*_ = **PM** + **I** − **P** is invertible and named as constraint inertia matrix. Then we get

(7) $$\begin{array}{r} \ddot{\text{q}}={\text{M}}_{c}^{-1}\left(\tau_{m}-\text{Ph}+\overset{∙}{\text{P}}\overset{∙}{\text{q}}\right) \end{array}$$

Substituting Equation (7) into Equation (4) yields the equation that can determine the contact forces

(8) $$\begin{array}{r} {\text{J}}_{c}^{⊤}\lambda =\left(\text{I}-\text{P}\right)\left[\text{M}{\text{M}}_{c}^{-1}\left(\tau_{m}-\text{Ph}+\overset{∙}{\text{P}}\overset{∙}{\text{q}}\right)+\text{h}\right]-\tau_{c} \end{array}$$

By replacing ***τ***_*m*_ and ***τ***_*c*_ with their original definitions in terms of ***τ***, Equation (8) becomes

(9) $$\begin{array}{r} {\text{J}}_{c}^{⊤}\lambda =\left(\text{I}-\text{P}\right)\left[-\left(\text{I}-\text{M}{\text{M}}_{c}^{-1}\text{P}\right)\left(\text{B}\tau -\text{h}\right)+\text{M}{\text{M}}_{c}^{-1}\overset{∙}{\text{P}}\overset{∙}{\text{q}}\right] \end{array}$$

It should be noted that the utility of Equation (7) is key for the derivation of the analytical impedance control law in section 3 and the reduction of optimization parameters in section 4 to ***τ*** instead of ${\begin{array}{cc} {}[{\ddot{\text{q}}}^{⊤} & \tau^{⊤}] \end{array}}^{⊤}$.

## 3. Cartesian Impedance Control in Motion Space

### 3.1. Applying Cartesian Impedance Control to Underactuated Systems

Since contact forces have been removed from the projected inverse dynamics of Equation (3), we can design a Cartesian impedance controller to achieve a desired motion performance in motion space without considering contact constraints. First, for the four stance legs case, only the torso requires desired motion, the torso is therefore considered an end-effector. The objective of Cartesian impedance control is to dictate the disturbance response of the robot. If a given Cartesian location **x** of the end-effector is subject to an external disturbance **F**_*x*_ we would like to prescribe the motion at **x** as

(10) $$\begin{array}{r} \Lambda_{d}\ddot{\text{e}}+{\text{D}}_{d}\overset{∙}{\text{e}}+{\text{K}}_{d}\text{e}={\text{F}}_{x} \end{array}$$

where **e** = **x** − **x**_*d*_ is the motion error between the current pose and the desired pose, **Λ**_*d*_, **D**_*d*_ and **K**_*d*_ are the desired Cartesian inertia, damping, and stiffness matrices that define the Cartesian impedance behavior. On the other hand, adding the external disturbance into the general dynamic equation Results in

(11) $$\begin{array}{r} \text{M}\ddot{\text{q}}+\text{h}=\text{B}\tau +{\text{J}}_{c}^{⊤}\lambda +{\text{J}}_{x}^{⊤}{\text{F}}_{x} \end{array}$$

where **J**_*x*_ is the Jacobian matrix mapping external forces/torques to joint space. Project Equation (11) into the motion space

(12) $$\begin{array}{r} \text{P}\text{M}\ddot{\text{q}}+\text{P}\text{h}=\text{P}\text{B}\tau +\text{P}{\text{J}}_{x}^{⊤}{\text{F}}_{x} \end{array}$$

Next, we employ **M**_*c*_ to transform Equation (12) to operational space. Equation (12) can be rewritten as

(13) $$\begin{array}{r} {\text{M}}_{c}\ddot{\text{q}}+\text{Ph}-\overset{∙}{\text{P}}\overset{∙}{\text{q}}=\text{PB}\tau +\text{P}{\text{J}}_{x}^{⊤}{\text{F}}_{x} \end{array}$$

Pre-multiplying by ${\text{J}}_{x}{\text{M}}_{c}^{-1}$ gives

(14) $$\begin{array}{r} {\text{J}}_{x}\ddot{\text{q}}+{\text{J}}_{x}{\text{M}}_{c}^{-1}\left(\text{Ph}-\overset{∙}{\text{P}}\overset{∙}{\text{q}}\right)={\text{J}}_{x}{\text{M}}_{c}^{-1}\text{PB}\tau +{\text{J}}_{x}{\text{M}}_{c}^{-1}\text{P}{\text{J}}_{x}^{⊤}{\text{F}}_{x} \end{array}$$

We replace ${\text{J}}_{x}\ddot{\text{q}}$ with $\ddot{\text{x}}-{\overset{∙}{\text{J}}}_{x}\overset{∙}{\text{q}}$ and multiply by $\Lambda_{c}={\left({\text{J}}_{x}{\text{M}}_{c}^{-1}\text{P}{\text{J}}_{x}^{⊤}\right)}^{-1}$

(15) $$\begin{array}{r} \Lambda_{c}\ddot{\text{x}}+\Lambda_{c}{\text{J}}_{x}{\text{M}}_{c}^{-1}\left(\text{Ph}-\overset{∙}{\text{P}}\overset{∙}{\text{q}}\right)-\Lambda_{c}{\overset{∙}{\text{J}}}_{x}\overset{∙}{\text{q}}=\Lambda_{c}{\text{J}}_{x}{\text{M}}_{c}^{-1}\text{PB}\tau +{\text{F}}_{x} \end{array}$$

where **Λ**_*c*_ is named as the constraint-consistent operational space inertia matrix. For the moment, we will assume full actuation **B** = **I**. In the latter, we will deal with the underactuation constraint separately. Then, if we replace all non-linear terms with **h**_*c*_ and ***τ*** with ${\text{J}}_{x}^{⊤}\text{F}$, we can compactly write the above equation as

(16) $$\begin{array}{r} \Lambda_{c}\ddot{\text{x}}+{\text{h}}_{c}=\text{F}+{\text{F}}_{x} \end{array}$$

where **F** is the operational forces due to actuation. In order to achieve the impedance response of Equation (10), we can define the control law as

(17) $$\begin{array}{r} \text{F}={\text{h}}_{c}+\Lambda_{c}{\ddot{\text{x}}}_{d}-\Lambda_{c}\Lambda_{d}^{-1}\left({\text{D}}_{d}\overset{∙}{\text{e}}+{\text{K}}_{d}\text{e}\right)+\left(\Lambda_{c}\Lambda_{d}^{-1}-\text{I}\right){\text{F}}_{x} \end{array}$$

We notice that if we sacrifice the ability to shape the inertia matrix by specifying **Λ**_*d*_ = **Λ**_*c*_ our control law simplifies to

(18) $$\begin{array}{r} \text{F}={\text{h}}_{c}+\Lambda_{c}{\ddot{\text{x}}}_{d}-{\text{D}}_{d}\overset{∙}{\text{e}}-{\text{K}}_{d}\text{e} \end{array}$$

The advantage of the above control law is that we do not need to measure the external disturbances **F**_*x*_ (e.g., with a force/torque sensor) to realize the impedance response.

On the other hand, by assigning **Λ**_*d*_ = **Λ**_*c*_, the impedance gains of Equation (10) can still be tuned by analytical techniques based on different configurations (see in Angelini et al., 2019), whereas HQP controllers rely on manual trial and error tuning.

**Remark**. *It should be noted that if* **J**_*x*_ *is rank deficient*, ${\text{J}}_{x}{\text{M}}_{c}^{-1}\text{P}{\text{J}}_{x}^{⊤}$ *is not invertible, and we cannot determine* $\Lambda_{c}={\left({\text{J}}_{x}{\text{M}}_{c}^{-1}\text{P}{\text{J}}_{x}^{⊤}\right)}^{-1}$. *This may happen, for example, for the case when the robot is trotting (only two point feet on the ground) or for a swing leg when the leg is in singularity configuration. In these cases we can approximate* **Λ**_*c*_ *by computing a singularity robust inverse of* ${\text{J}}_{x}{\text{M}}_{c}^{-1}\text{P}{\text{J}}_{x}^{⊤}$, *for example, by Singular Value Decomposition (SVD) with zero or near-zero eigenvalues removed*.

### 3.2. Imposing Underactuation Constraints

The previous derivation of the control law (Equation 18) for Cartesian impedance control is based on an assumption of **B** = **I**. In fact, a quadruped robot is a typical underactuated system, i.e., the definition of **B** in Equation (2) is not an identity matrix. We need to satisfy the additional underactuation constraint

(19) $$\begin{array}{r} \tau_{m}=\text{B}\tau_{m} \end{array}$$

Our control law of Equation (18) is defined in operational space. Although in general ${\text{J}}_{x}^{⊤}\text{F}\neq \text{B}{\text{J}}_{x}^{⊤}\text{F}$, we may still be able to satisfy (19) by adding constraint forces in constraint space

(20) $$\begin{array}{r} \tau_{m}={\text{J}}_{x}^{⊤}\text{F}+\left(\text{I}-\text{P}\right)\tau_{0}=\text{B}{\text{J}}_{x}^{⊤}\text{F}+\text{B}\left(\text{I}-\text{P}\right)\tau_{0} \end{array}$$

and we can solve for ***τ***_0_

(21) $$\begin{array}{r} \left(\text{I}-\text{B}\right){\text{J}}_{x}^{⊤}\text{F}=-\left(\text{I}-\text{B}\right)\left(\text{I}-\text{P}\right)\tau_{0} \end{array}$$

(22) $$\begin{array}{r} \tau_{0}=-{\left[\left(\text{I}-\text{B}\right)\left(\text{I}-\text{P}\right)\right]}^{+}\left(\text{I}-\text{B}\right){\text{J}}_{x}^{⊤}\text{F} \end{array}$$

Subsequently, the Cartesian impedance control law in motion space with consideration of underactuation becomes

(23) $$\begin{array}{r} \tau_{m}={\text{J}}_{x}^{⊤}\text{F}-\left(\text{I}-\text{P}\right){\left[\left(\text{I}-\text{B}\right)\left(\text{I}-\text{P}\right)\right]}^{+}\left(\text{I}-\text{B}\right){\text{J}}_{x}^{⊤}\text{F} \end{array}$$

Furthermore, as described in Mistry and Righetti (2012), it could be simplified to

(24) $$\begin{array}{r} \tau_{m}={\left(\text{PB}\right)}^{+}{\text{J}}_{x}^{⊤}\text{F} \end{array}$$

where **F** is defined in Equation (18).

**Figure 2:**
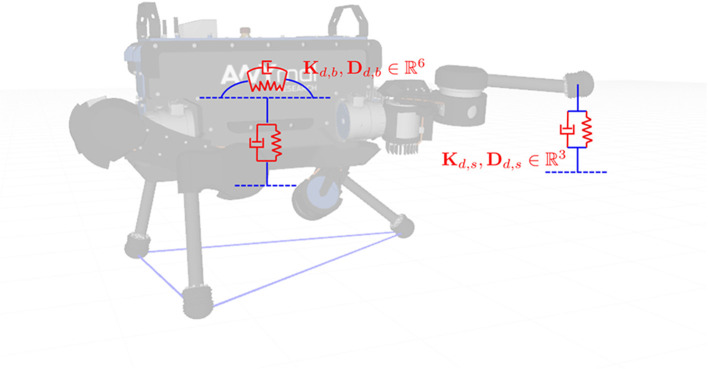
The base pose and the swing foot position are controlled by two Cartesian impedance controllers.

The above Cartesian impedance controller could also be applied to swing legs by replacing **J**_*x*_ with corresponding Jacobian matrix of a swing foot. If we define the Jacobian matrix of torso and one swing leg as **J**_*b*_ and **J**_*s*_, respectively, the only difference is ${\text{J}}_{s}\in {\mathbb{R}}^{3\times \left(n+6\right)}$ due to the point foot while ${\text{J}}_{b}\in {\mathbb{R}}^{6\times \left(n+6\right)}$. Furthermore, let ${\text{F}}_{b}\in {\mathbb{R}}^{6}$ and ${\text{F}}_{s}\in {\mathbb{R}}^{3}$ represent the control law of Equation (18) for torso and swing feet, resulting in the torque command of motion space as follows

(25) $$\begin{array}{r} \tau_{m}={\left(\text{PB}\right)}^{+}\left({\text{J}}_{b}^{⊤}{\text{F}}_{b}+\overset{n_{legs}}{\underset{i=0}{\sum }}\omega {\text{J}}_{s,i}^{⊤}{\text{F}}_{s,i}\right) \end{array}$$

where weight ω is 1 when leg *i* is in swing, and 0 otherwise. Figure 2 depicts the case of two Cartesian impedance controllers applied to base and one foot, respectively.

### 3.3. Model Error Estimation and Compensation

One of the benefits of Cartesian impedance control is that the impedance response of Equation (10) provides us with a way to estimate the disturbances using motion errors

(26) $$\begin{array}{r} {\hat{\text{F}}}_{x}=\Lambda_{d}\ddot{\text{e}}+{\text{D}}_{d}\overset{∙}{\text{e}}+{\text{K}}_{d}\text{e} \end{array}$$

It should be noted that ${\hat{\text{F}}}_{x}$ contains external disturbances as well as model errors. Considering the existing model error, the control law of Equation (18) becomes

(27) $$\begin{array}{r} \text{F}={\hat{\text{h}}}_{c}+{\hat{\Lambda }}_{c}{\ddot{\text{x}}}_{d}-{\text{D}}_{d}\overset{∙}{\text{e}}-{\text{K}}_{d}\text{e} \end{array}$$

where ${\hat{\text{h}}}_{c}$ and ${\hat{\Lambda }}_{c}$ denote the approximate items in computation. Substituting Equation (27) into operational space dynamics of Equation (16) yields

(28) $$\begin{array}{r} {\hat{\Lambda }}_{c}\ddot{\text{e}}+{\text{D}}_{d}\overset{∙}{\text{e}}+{\text{K}}_{d}\text{e}=\left({\text{h}}_{c}-{\hat{\text{h}}}_{c}\right)+\left(\Lambda_{c}-{\hat{\Lambda }}_{c}\right)\ddot{\text{x}}+{\text{F}}_{x} \end{array}$$

Further, the above equation implies ${\hat{\text{F}}}_{x}=\left({\text{h}}_{c}-{\hat{\text{h}}}_{c}\right)+\left(\Lambda_{c}-{\hat{\Lambda }}_{c}\right)\ddot{\text{x}}+{\text{F}}_{x}$ where not only external disturbances are involved, but also model errors. In the case where small external disturbances exist, Equation (28) can be used to estimate model errors.

This feature is useful for legged robots to carry unknown objects. If we feed the model error (e.g., caused by adding an object of unknown mass onto the robot) back into the control law of Equation (18), the position errors due to model errors will be removed. Therefore, the robot may carry unknown objects by compensating for estimated model errors. The control law in motion space with model-error compensation becomes

(29) $$\begin{array}{r} \text{F}={\hat{\text{h}}}_{c}+{\hat{\Lambda }}_{c}{\ddot{\text{x}}}_{d}-{\text{D}}_{d}\overset{∙}{\text{e}}-{\text{K}}_{d}\text{e}+{\hat{\text{F}}}_{x} \end{array}$$

where ${\hat{\text{F}}}_{x}$ is constant by computing Equation (28) once at the beginning of switching on model error compensation.

By contrast, HQP controllers cannot use motion errors to estimate disturbances because the gains of HQP controllers have different scaling. We will verify this point in the experimental section.

## 4. Quadratic Programming

The control scheme outlined above does not yet impose physical feasibility on the contact forces and joint torques. In this section, these constraints are introduced, and we present our quadratic programming approach to handle them.

### 4.1. Physical Constraints

The contact forces at the feet should be sufficient to prevent the separation or sliding of the contact. Additionally, motor torques typically have saturation values. To formalize the contact force constraint, the force at each foot is divided into tangential components λ_*x*_ and λ_*y*_, and normal component λ_*z*_. This leads to the following constraints:

- Unilateral constraint to avoid loss of contact
  (30) $$\begin{array}{r} \lambda_{z}\geq 0 \end{array}$$
- Friction cone constraint to avoid slipping
  (31) $$\begin{array}{r} \mu \lambda_{z}\geq \sqrt{\lambda_{x}^{2}+\lambda_{y}^{2}} \end{array}$$

where μ is the friction coefficient at the contact point.

The saturation of torque command gives.

(32) $$\begin{array}{r} \tau_{\text{min}}\leq \tau \leq \tau_{\text{max}} \end{array}$$

### 4.2. Optimization Formulation

Our previous work (Xin et al., 2018) minimized a quadratic cost function of the torque commands, thereby minimizing the torques.

(33) $$\begin{array}{r} \underset{\tau }{\text{minimize}}\;\frac{1}{2}||\tau |{|}_{2}^{2} \end{array}$$

where ***τ*** = ***τ***_*m*_ + ***τ***_*c*_. To track trajectories and to deal with external disturbances, the Cartesian impedance controller (25) defines the motion space torque command ***τ***_*m*_. This partial command is included in the optimization using the linear equality constraint:

(34) $$\begin{array}{r} \text{PB}\tau =\tau_{m} \end{array}$$

The friction forces are computed from the optimization variable ***τ*** based on (9) and simplified to be

(35) $$\begin{array}{r} \lambda =\eta +\rho \end{array}$$

where $\eta =-{\left({\text{J}}_{c}^{⊤}\right)}^{+}\left(\text{I}-\text{P}\right)\left(\text{I}-\text{M}{\text{M}}_{c}^{-1}\text{P}\right)\text{B}\tau$ and $\rho ={\left({\text{J}}_{c}^{⊤}\right)}^{+}\left(\text{I}-\text{P}\right)\left[\left(\text{I}-\text{M}{\text{M}}_{c}^{-1}\text{P}\right)\text{h}+\text{M}{\text{M}}_{c}^{-1}\overset{∙}{\text{P}}\overset{∙}{\text{q}}\right]$. Then we can transform the unilateral constraint and friction cone constraint to be written in terms of decision variable ***τ***. The quadratic programming problem is now formulated as:

(36) $$\begin{array}{r} \begin{array}{cc} \underset{\tau }{\text{minimize}} & \frac{1}{2}||\tau |{|}_{2}^{2}\\ \text{subject to} & \text{PB}\tau =\tau_{m}\\ \eta_{z}^{i}+\rho_{z}^{i}\geq 0\\ \mu \left(\eta_{z}^{i}+\rho_{z}^{i}\right)\geq \sqrt{{\left(\eta_{x}^{i}+\rho_{x}^{i}\right)}^{2}+{\left(\eta_{y}^{i}+\rho_{y}^{i}\right)}^{2}}\\ \tau_{\text{min}}\leq \tau \leq \tau_{\text{max}} \end{array} \end{array}$$

where the index *i* signifies the *i*th stance leg.

### 4.3. Adding Trade-Off Between Multiple Constraints

In practice, there may not be a physically feasible solution that achieves both the desired motion and impedance behavior. For example, tangential contact forces may increase due to the desired acceleration for walking forward, causing contact forces to violate the friction cone. To avoid a need for replanning the desired motion, we propose a trade-off between the constraints. The inequality constraints cannot be traded-off because they enforce physical limitations and stability of the system. On the other hand, the equality constraint enforces the operational space tasks. We will sacrifice motion performance to ensure feasibility of the physical (inequality) constraints when there is no possibility of finding a solution that satisfies both types of constraints. Here, we employ a trade-off of the trajectory tracking control to seek a compromise in low level control. The trade-off is implemented by moving the equality constraint into the cost function to compose a least squares optimization.

(37) $$\begin{array}{r} \begin{array}{cc} \underset{\tau }{\text{minimize}} & \frac{1}{2}||\text{PB}\tau -\tau_{m}|{|}_{2}^{2}\\ \text{subject to} & \eta_{z}^{i}+\rho_{z}^{i}\geq 0\\ \mu \left(\eta_{z}^{i}+\rho_{z}^{i}\right)\geq \sqrt{{\left(\eta_{x}^{i}+\rho_{x}^{i}\right)}^{2}+{\left(\eta_{y}^{i}+\rho_{y}^{i}\right)}^{2}}\\ \tau_{\text{min}}\leq \tau \leq \tau_{\text{max}} \end{array} \end{array}$$

The above formulation means that the QP optimization will try to find the admissible torques that are as close as possible to the torque command of motion space required for the desired motion performance. It should be noted that if the QP needs to trade-off the motion performance it will change the swing trajectory more than the torso trajectory, as the torso contributes more to contact forces than the swing leg. Alternatively, we could add a weighting matrix to balance the modification of different parts if it is necessary. However, we would then need to tune the weighting matrix in practice. Therefore, we recommend using a null space projection of the swing legs to project the cost function, in order to enforce accurate swing leg performance. After the QP provides the optimized torque command based on the torso motion, we will add the dynamic consistent torques for the swing legs. The QP in the null space of the swing legs becomes

(38) $$\begin{array}{r} \begin{array}{cc} \underset{\tau }{\text{minimize}} & \frac{1}{2}||{\text{N}}_{s}\left(\text{PB}\tau -\tau_{m}\right)|{|}_{2}^{2}\\ \text{subject to} & \eta_{z}^{i}+\rho_{z}^{i}\geq 0\\ \mu \left(\eta_{z}^{i}+\rho_{z}^{i}\right)\geq \sqrt{{\left(\eta_{x}^{i}+\rho_{x}^{i}\right)}^{2}+{\left(\eta_{y}^{i}+\rho_{y}^{i}\right)}^{2}}\\ \tau_{\text{min}}\leq \tau \leq \tau_{\text{max}} \end{array} \end{array}$$

where ${\text{N}}_{s}=\text{I}-{\text{J}}_{s}^{+}{\text{J}}_{s}$, **J**_*s*_ = **J**_*s*, 1_ for static walking gait, ${\text{J}}_{s}=\left(\begin{array}{c} {\text{J}}_{s,1}\\ {\text{J}}_{s,2} \end{array}\right)$ for the gaits with only two supporting legs, such as trotting gait, and **J**_*s*_ = **0** for four stance legs case. The final torque command will be

(39) $$\begin{array}{r} \tau =\tau^{*}+\left(\text{I}-{\text{N}}_{s}\right)\tau_{m} \end{array}$$

**Figure 3:**
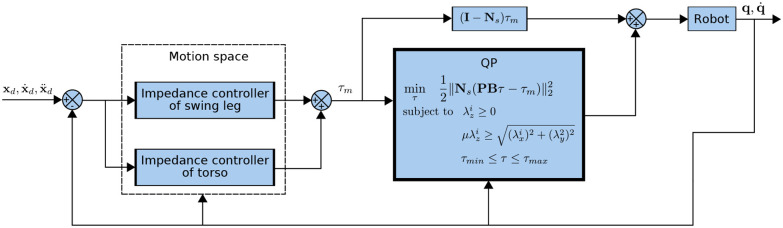
An overview of the proposed locomotion controller with Cartesian impedance control and quadratic programming subject to physical constraints.

The controller proposed above is represented as a block diagram in Figure 3.

**Remark**. *One notices that the friction cone constraints are quadratic constraints. Therefore, the quadratic programming of Equation (38) is quadratically constrained quadratic programming. In order to use standard active set methods, we approximate the friction cone with a linearized 4-edge pyramid. This results in a linearly constrained quadratic programming problem, which is readily solved (Goldfarb and Idnani, 1983)*.

## 5. Experiments and Discussion

To validate our proposed controller, three sets of experiments were performed: a comparison the HQP controller, model error compensation, and walking on challenging terrains. This section treats each of the experiments in turn, and provides a setup description, results, and discussion for all of them.

The experiments have been performed using ANYmal, a torque-controlled quadruped robot made by ANYbotics. The robot weights ~35 kg and has 12 joints actuated by the Series Elastic Actuator (SEA). The control cycle runs at 400Hz. The on-board computer running controllers has an Intel 4th generation (Haswell ULT) i7-4600U (1.4–2.1GHz) processor and two HX316LS9IBK2/16 DDR3L memory cards. Experiment videos can be found in the Supplementary Video 1.

### 5.1. Comparison With HQP Controller

To show the efficacy of our proposed controller, we compare it with an HQP-computer. This comparison considers both the computational efficiency and the capacity to estimate external disturbances. Our implementation of the HQP controller follows the original framework from De Lasa et al. (2010), adding a dimension-reduction to that framework in order to enhance computational efficiency. This dimension-reduction, proposed in Herzog et al. (2016) removes the motor-torque from the decision variables by using the equations of motion of the robot. The remaining decision variables are the torso and joint accelerations and the contact forces. An active-set QP solver was employed to solve QPs in both controllers.

#### 5.1.1. Computational Efficiency

To evaluate the computational efficiency of the proposed whole-body controller against the HQP-controller, we compare their execution time on the robot hardware. A comparison of average computation times while executing a fixed static walking gait is provided in Table 1.

**Table 1:** The computation efficiency comparison between proposed controller (P.C.) and HQP controller during static walking.

**Controller**	**Phase**	**τ_*m*_**	**QP_**1**_**	**N_**1**_**	**QP_**2**_**	**N_**2**_**	**QP_**3**_**	**N_**3**_**	**QP_**4**_**	**Total (m/s)**
P.C.	Stance	0.203	0.064							0.267
	Swing	0.147	0.095							0.242
HQP	Stance		0.09	0.124	0.1	0.024	0.09			0.428
	Swing		0.075	0.081	0.098	0.02	0.072	0.025	0.079	0.45

*The results are the means of 2,000 samples, respectively. HQP controller needs to compute two null-space projectors N_i_ and solve three QPs during Stance phase, whereas one more QP is added for swing leg control during Swing phase. The proposed controller only needs to compute analytical Cartesian impedance control ***τ***_m_ and solve one QP. The computation time of HQP is almost doubled compared to the proposed controller either in Stance phase or in Swing phase*.

The computation time analysis shows that our controller is faster than the HQP controller. This is expected since our controller only requires one QP although it computes a few pseudo-inverses of matrices. By contrast, HQP needs to compute more QPs and also needs to compute null-space projectors which include pseudo-inverses as well. In fact, HQP needs to solve at least two QPs for dynamic feasibility and trajectory tracking tasks. In order to save energy, a third QP has to be added to minimize torque commands. Usually, those are the three fundamental QPs required by HQP controllers (Bellicoso et al., 2019). For walking gaits, if we separate swing legs and torso into different prioritized tasks, one more QP has to be solved. In this experiment, the HQP controller solved three QPs for the four-legged stance phase and four QPs during a one leg swing. In Herzog et al. (2016), the first QP is solved by pseudo-inverse when moving friction cone constraints into a lower priority, which aims to save computation time. But, based on Table 1, the computation time of HQP is still greater than the proposed controller, even omitting the computation time of QP_1_. Furthermore, Herzog et al. (2016) also suggests parallelizing the computations of null-space projectors and QPs. In that case, the total computation time of HQP will be the sum of solving QPs, which is close to our controller's according to Table 1. What's more, as pointed out in Escande et al. (2014) and Herzog et al. (2016), the size of decision variables can be reduced over the levels of hierarchies by additional SVD, which could potentially improve the computation efficiency of HQP even further. Readers should keep in mind that we did not employ that technique in the comparison of Table 1.

#### 5.1.2. External Disturbance Estimation

The ability of the two controllers to estimate external disturbances is assessed on the robot by comparing their response to similar force perturbations while standing still. The operator pushed the torso along the vertical axis using about 30N force, and the recorded position displacements are used to estimate the external perturbation received by the robot. As would happen in practice, we only use the multiplication of **K**_*d*_ and displacement errors as the estimated disturbance instead of the entire Equation (28) since accelerations and velocities are noisy.

**Figure 4:**
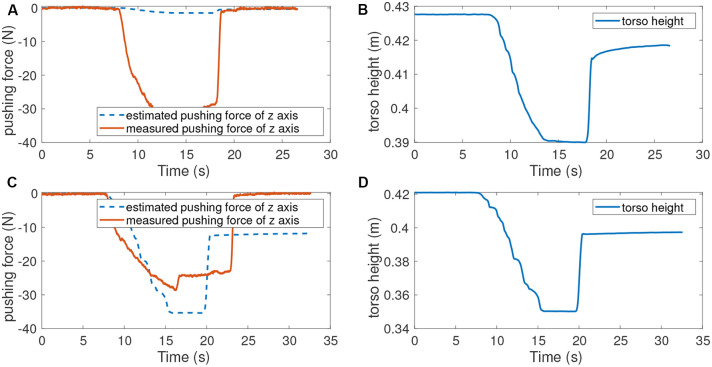
The pushing forces and torso positions when pushing the robot down from standing still. **(A)** Measured and estimated pushing forces when running HQP controller. **(B)** Torso height during pushing with HQP running. **(C)** Measured and estimated pushing forces when running the proposed controller. **(D)** Torso height during pushing with the proposed controller running. The similarity in the response of both controllers shows that the effective torso stiffness is roughly equal, despite the large difference in gains used. HQP cannot estimate disturbance forces whereas the estimated force of our controller is roughly close to the measured force.

Figure 4 shows the measured pushing forces and torso heights, which have been obtained using the gains reported in Table 2. The position errors caused by pushing forces are similar between the two controllers, which means they produce approximately the same effective stiffness. The force estimated by the proposed controller is **K**_*d,z*_**e**_*z*_ ≈ 35*N*. This estimation is close to the measured pushing force 30N. However, the relationship between the effective stiffness and the controller gains is not straightforward for the HQP-controller. For this controller, the gains map from an error to a desired acceleration, not to a desired force, as is the case for our proposed controller. As a result, the **K**_*d*_ gains of the HQP controller are much smaller than the ones of the proposed controller, meaning the HQP estimated force is 1.6N which is much lower than the received external perturbation. A conversion factor could be found, either based on the equations of motion or using experiments, such as this one. However, the conversion factor would depend on the robot configuration, which means the HQP-controller would require extensive tuning to be able to perform disturbance estimation. Therefore, the proposed controller is more suitable for performing the forces estimation for the compensation of constant external perturbations, such as the additional load carried by the robot.

**Table 2:** Gains for HQP controller and the proposed controller used in the stiffness comparison experiment.

**Parameter**	**Symbol**	**HQP**	**Proposed controller**
Stiffness gains for torso	***K***_***d***_	diag(40,40,40,60,60,60)	diag(1000,800,500,200,200,200)
Damping gains for torso	***D***_***d***_	diag(9,9,9,7,7,7)	diag(250,250,90,10,10,10)

*Although the gains are so different in the two controllers especially for torso control, the torso showed similar stiffness performance*.

### 5.2. Model Error Compensation

Legged robots may be deployed to move small loads which may not always be known; thus, it is important to adapt the robot behavior to maintain stable and efficient locomotion. Equation (29) describes how the proposed controller may be modified to use the external perturbation estimation to perform a model error compensation. For the experiment, the robot was loaded with a 10 kg mass and walked with and without activating the model compensation, which has been enabled after about 23 s. Table 3 contains the gains of the proposed controller in the experiment. The recorded video for this experiment can be found in the Supplementary Material.

**Table 3:** Gains of the proposed controller during carrying weight experiment.

**Parameter**	**Symbol**	**Proposed controller**
Stiffness gains for torso	***K***_***d***_	diag(1000,800,1000,200,200,200)
Damping gains for torso	***D***_***d***_	diag(250,250,250,10,10,10)
Stiffness gains for swing foot	***K***_***d***_	diag(250,250,250)
Damping gains for swing foot	***D***_***d***_	diag(20,20,20)

**Figure 5:**
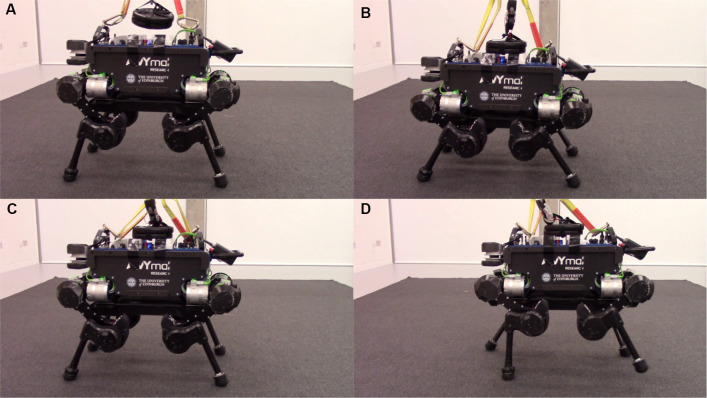
Snapshots of carrying 10 kg weight with model error compensation. **(A)** Before adding the weight. **(B)** After adding the weight, the torso went down. **(C)** The torso went up after enabling model error compensation. **(D)** Walking with the object with model error compensation.

**Figure 6:**
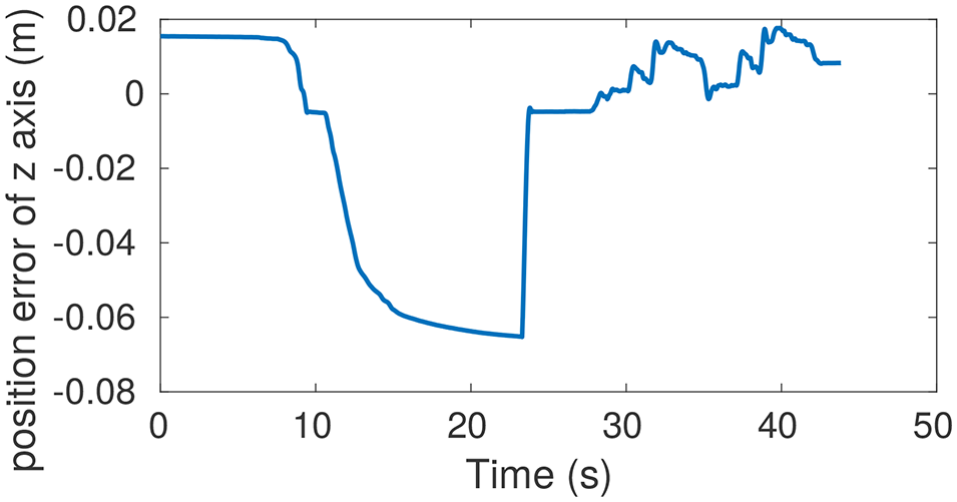
Measured torso height during carrying 10 kg weight. At 10 s, the weight is slowly lowered onto the robot, resulting in an increasing position error. At 23 s the force compensation is enabled, after which the robot takes two steps. The small difference between the initial and final position error shows the efficacy of the disturbance compensation.

Figure 5 shows the snapshots from the experiments of adding weight to walking with it. Figure 6 is the recorded position error along the *z* axis during the experiment. After enabling the compensation, the torso immediately rose as shown in Figure 6 and continued its locomotion with a reduced position error. It should be noted that the estimated model error is about 65N. A bias of 35N has been added in this experiment to account for friction and other uncertainties of a real robot. The results demonstrate that the position error can be reduced by model error compensation when carrying unknown objects, which is useful for deployment of legged robots in th real world. However, an important upgrade required to deploy this feature in a real scenario is continuous real-time model adaptation, which has currently been left as future work to develop.

### 5.3. Walking on Challenging Terrains

**Figure 7:**
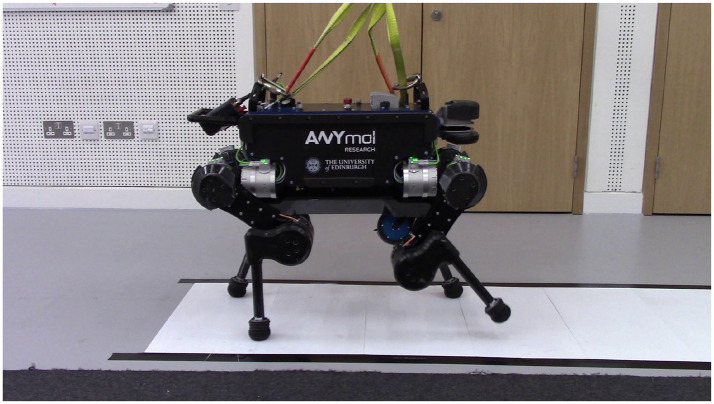
ANYmal walking on slippery synthetic ice. The estimated friction coefficient between ANYmal's feet and the synthetic ice is 0.22. The friction coefficient in our controller needs to be ≤0.2 in order to avoid slipping on the synthetic ice.

**Figure 8:**
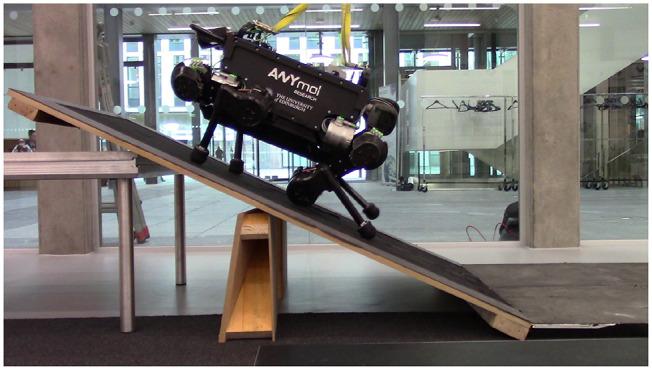
ANYmal climbing up on a 30° slope.

We tested the proposed controller for its ability to tackle challenging terrains. We tested walking on a low friction surface (PTFE Sheets, fiction coefficient of 0.22 as shown in Figure 7), and the feasibly of climbing a significant incline (30° slope in Figure 8). As perception is not the focus of this work, the friction coefficient and slope angle parameters, to be used by the controller in the optimization stage, are set manually. Locomotion performance for these tasks may be seen in the accompanying video.

We did not alter the step planning in our experiment, but rather used the default ANYbotics static gait planner. This means the planned locomotion trajectory was not optimized for our particular tasks or evaluated for feasibility. Thus, during execution, if the controller does not trade-off trajectory tracking and constraint satisfaction, the robot may slip due to the inability of the QP finding a feasible solution. We further evaluate this trade-off by analyzing trajectory tracking errors when statically walking on flat terrain, while varying the friction coefficient parameter used by the controller.

**Figure 9:**
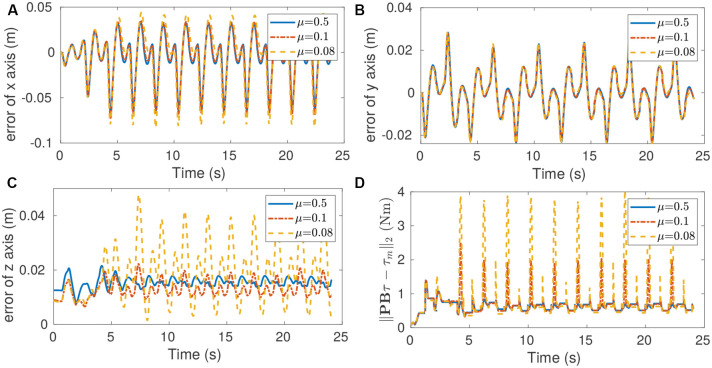
Tracking performance when walking with a static gait on high-traction flat terrain for different values of the friction coefficient used by the controller. **(A)** Position tracking error of *x* direction. **(B)** Position tracking error of y direction. **(C)** Position tracking error of *z* direction. **(D)** Deviation between **PB*****τ*** and ***τ***_*m*_. The increasing errors for small friction coefficients show the trade-off in tracking performance made to guarantee feasibility of the friction cone constraint. In particular, a behavior emerges in which the controller deviates from the target motion in *z* direction, in order to increase normal contact forces when walking with a small friction coefficient. The trade-off between tracking error and constraint satisfaction is also visible in the increasing deviation between **PBτ** and ***τ***_*m*_.

As shown in Figure 9, the trajectory tracking error along the *z* direction has an occasional sharp rise particularly when the friction coefficient μ is equal to 0.1 and 0.08. This indicates that trajectory tracking performance is sacrificed in order to maintain the friction cone constraint. We also see that the cost function value of QP increases, due to the increase of trajectory tracking error. In theory, the value of cost function should be zero if the trade-off is not triggered. The model error explains why the deviation between **PB*****τ*** and ***τ***_*m*_ is not equal to zero even when μ = 0.5. The controller in our previous work (Xin et al., 2018) does not have this trade-off feature, and this is an improvement over our previous work.

## 6. Conclusions

A semi-analytical locomotion controller incorporating an analytical Cartesian impedance controller and a QP optimization for quadruped robots is presented in the paper. Cartesian impedance controllers can track desired end-effector trajectories and can also be used to estimate external disturbances. The disturbance estimation is applied to model error compensation in the case of making a robot carry unknown objects. The QP optimization guarantees physical feasibility by sacrificing trajectory tracking if the torque command required to track the desired trajectory does not satisfy physical constraints. The benefit of this trade-off strategy enables the robot to walk on synthetic ice and a 30° ramp. Besides, the computation time is verified to be less than other fully optimization-based whole-body controllers since we only need to solve one QP with less decision variables compared to other controllers. Future work will focus on applying this control framework to dynamic gaits, such as trotting.

## Data Availability Statement

All datasets generated for this study are included in the article/Supplementary Material.

## Author Contributions

GX, H-CL, and MM significantly contributed to the development of the presented approach. GX, WW, and CT added the least-square optimization to the framework. All authors have contributed to the writing and they have reviewed the submitted version.

## Conflict of Interest

The authors declare that the research was conducted in the absence of any commercial or financial relationships that could be construed as a potential conflict of interest.

## Supplementary Material

Supplementary Video 1 Model error compensation and walking on challenging terrains of a quadruped robot.
